# Supplementary material for: Rationale for the shielding policy for clinically vulnerable people in the UK during the COVID-19 pandemic: a qualitative study
Source: BMJ Open. 2023 Aug 4;13(8):e073464. doi: 10.1136/bmjopen-2023-073464 (PMC10407356; doi:10.1136/bmjopen-2023-073464)
Supplement: Supplementary data [file bmjopen-2023-073464supp001.pdf]

Appendix **EVITE Immunity Phase 1 – WP1 QUESTIONS FOR STAKEHOLDER CONSULTATION**

This consultation is about understanding the rationale for the shielding programme in Wales for patients clinically extremely vulnerable to COVID-19 infection.

1. What was the objective of the shielding programme?
    - To reduce mortality among vulnerable people
    - To limit pressure on the NHS
    - To reduce infection rates/morbidity
    - Were there multiple objectives? If so, was one more important that the others?
    - Did the objectives change over time?

2. How was the shielding programme designed to work?
    - Change in behaviour of people at risk (staying home/avoiding contact)
    - Change in behaviour of other household members (staying home/avoiding contact)
    - Adaptations made by employers (allowing people to work from home, furloughing staff)
    - Increasing awareness of those clinically vulnerable aka the shielding population
    - Increasing awareness of risk among the wider public
    - Was there any change over time in how the programme was designed to work
3. What was needed to make it work?
    - Identifying people at risk – datasets, risk algorithms, clinical judgement
    - Information/communication with people on the list (letter vs text mechanism and accessibility of these)
    - Support/advice from medical staff (GPs or consultants managing patients' conditions)
    - Support/advice from carers of those identified as clinically vulnerable
    - Food parcels
    - Benefits/compensation for people unable to work
    - Have all these been in place? Has anything changed over time?

4. How was the shielding programme developed?
    - Where did the idea come from?
    - Who was involved in developing the programme?
    - Who made the decisions?
    - What contact was there with partners across the UK?
    - How did the shielding programme in Wales relate to England?
    - as there consensus among all involved about the objectives of the programme and what form it should take?

5. How has the shielding programme been reviewed/monitored?
    - What information has been gathered?
    - What has changed as a result?
