# Supplementary material for: Rationale for the shielding policy for clinically vulnerable people in the UK during the COVID-19 pandemic: a qualitative study
Source: BMJ Open. 2023 Aug 4;13(8):e073464. doi: 10.1136/bmjopen-2023-073464 (PMC10407356; doi:10.1136/bmjopen-2023-073464)
Supplement: Author's manuscript [file bmjopen-2023-073464.draft_revisions.pdf]

BMJ Open is committed to open peer review. As part of this commitment we make the peer review history of every article we publish publicly available.

When an article is published we post the peer reviewers' comments and the authors' responses online. We also post the versions of the paper that were used during peer review. These are the versions that the peer review comments apply to.

The versions of the paper that follow are the versions that were submitted during the peer review process. They are not the versions of record or the final published versions. They should not be cited or distributed as the published version of this manuscript.

BMJ Open is an open access journal and the full, final, typeset and author-corrected version of record of the manuscript is available on our site with no access controls, subscription charges or pay-per-view fees (http://bmjopen.bmj.com).

If you have any questions on BMJ Open's open peer review process please email info.bmjopen@bmj.com

BMJ Open

Rationale for the shielding policy for clinically vulnerable people in the UK during the COVID-19 pandemic: a qualitative study

Journal:	*BMJ Open*
Manuscript ID	bmjopen-2023-073464
Article Type:	Original research
Date Submitted by the Author:	07-Mar-2023

Complete List of Authors:	Porter, Alison; Swansea University, Swansea University Medical School<br>Akbari, Ashley; Swansea University Medical School,<br>Carson-Stevens, A; Cardiff University<br>Dale, Jeremy; University of Warwick, Warwick Medical School<br>Dixon, Lucy; Public contributor<br>Edwards, Adrian; Cardiff University,<br>Griffiths, Lesley; Public Contributor<br>John, Ann; Swansea University, Swansea University Medical School<br>Jolles, Stephen; Cardiff University,<br>Kingston, Mark; Swansea University, Medicine<br>Lyons, Ronan; University of Wales Swansea, Swansea Clinical School<br>Morgan, Jennifer; NHS Wales, Delivery Unit<br>Sewell, Bernadette; Swansea University, College of Human and Health Sciences<br>Williams, Victoria; Swansea University, Swansea University Medical School<br>Snooks, Helen; Swansea University, Medicine

Keywords:	Public health < INFECTIOUS DISEASES, QUALITATIVE RESEARCH, PUBLIC HEALTH, COVID-19

SCHOLARONE™
Manuscripts

I, the Submitting Author has the right to grant and does grant on behalf of all authors of the Work (as defined in the below author licence), an exclusive licence and/or a non-exclusive licence for contributions from authors who are: i) UK Crown employees; ii) where BMJ has agreed a CC-BY licence shall apply, and/or iii) in accordance with the terms applicable for US Federal Government officers or employees acting as part of their official duties; on a worldwide, perpetual, irrevocable, royalty-free basis to BMJ Publishing Group Ltd ("BMJ") its licensees and where the relevant Journal is co-owned by BMJ to the co-owners of the Journal, to publish the Work in this journal and any other BMJ products and to exploit all rights, as set out in our licence.

The Submitting Author accepts and understands that any supply made under these terms is made by BMJ to the Submitting Author unless you are acting as an employee on behalf of your employer or a postgraduate student of an affiliated institution which is paying any applicable article publishing charge ("APC") for Open Access articles. Where the Submitting Author wishes to make the Work available on an Open Access basis (and intends to pay the relevant APC), the terms of reuse of such Open Access shall be governed by a Creative Commons licence – details of these licences and which Creative Commons licence will apply to this Work are set out in our licence referred to above.

Other than as permitted in any relevant BMJ Author's Self Archiving Policies, I confirm this Work has not been accepted for publication elsewhere, is not being considered for publication elsewhere and does not duplicate material already published. I confirm all authors consent to publication of this Work and authorise the granting of this licence.

For peer review only

Title	Rationale for the shielding policy for clinically vulnerable people in the UK during the COVID-19 pandemic: a qualitative study
Authors' names	Alison Porter (0000-0002-3408-7007), Ashley Akbari, Andrew Carson-Stevens, Jeremy Dale, Lucy Dixon, Adrian Edwards, Bridie Evans, Lesley Griffiths, Ann John, Stephen Jolles, Ronan Lyons, Mark Kingston, Jennifer Morgan, Bernadette Sewell, Anthony Whiffen, Victoria Williams, Helen Snooks
Address for each author	Swansea University Medical School, ILS2, Singleton Park, Swansea SA2 8PP, UK Alison Porter Associate Professor of Health Services Research; Swansea University Medical School Ashley Akbari Senior Research Manager HDR-UK; Cardiff University UK Andrew Carson-Stevens Professor of Primary Care and Safety; Warwick University Medical School UK Jeremy Dale Professor of Primary Care; C/o Swansea University Medical School UK Lucy Dixon public representative; Cardiff University UK Adrian Edwards Professor of General Practice; Swansea University Medical School UK Bridie Evans Research Officer; C/o Swansea University Medical School UK Lesley Griffiths public representative; Swansea University Medical School UK Ann John Professor of Public Health Psychiatry; Cardiff University UK Stephen Jolles Professor of Clinical Immunology; Swansea University Medical School UK Mark Kingston Research Officer; Swansea University Medical School UK Jane Lyons Research Officer & Data Scientist ; Swansea University Medical School UK Ronan Lyons Professor of Public Health and Informatics; NHS Wales Delivery Unit UK Jennifer Morgan Operational Research and Modelling Development Manager; Swansea University Medical School UK Bernadette Sewell Health Economist; Administrative Data Research Unit, Welsh Government, UK Anthony Whiffen; Swansea University Medical School UK Victoria Williams Research Officer; Swansea University Medical School UK Helen Snooks Professor of Health Services Research.
Corresponding author	Alison Porter a.m.porter@swansea.ac.uk

Copyright: The Corresponding Author has the right to grant on behalf of all authors and does grant on behalf of all authors, an exclusive licence (or non exclusive for government employees) on a worldwide basis to the BMJ Publishing Group Ltd to permit this article (if accepted) to be published in BMJ editions and any other BMJPGL products and sublicences such use and exploit all subsidiary rights, as set out in our licence.

For peer review only - http://bmjopen.bmj.com/site/about/guidelines.xhtml

ABSTRACT

Introduction

Shielding aimed to protect those predicted to be at highest risk from COVID-19 and was uniquely implemented in the United Kingdom during the first year of the pandemic, from March 2020. As the first stage in the EVITE Immunity evaluation, we generated a logic model to describe the programme theory underlying the shielding intervention.

Design and participants

We reviewed published documentation on shielding to develop an initial draft of the logic model. We then discussed this draft during interviews with 13 key stakeholders involved in putting shielding into effect in Wales and England. Interviews were recorded, transcribed and analysed thematically to inform a final draft of the logic model.

Results

The shielding intervention was a complex one, introduced at pace by multiple agencies working together. We identified three core components: agreement on clinical criteria; development of the list of people appropriate for shielding; and communication of shielding advice. In addition, there was a support programme, available as required to shielding people, including food parcels, financial support, and social support. The predicted mechanism of change was that people would isolate themselves and so avoid infection, with the primary intended outcome being reduction in mortality in the shielding group. Unintended impacts included negative impact on mental and physical health and wellbeing. Details of the intervention varied slightly across the home nations of the UK, and were subject to minor revisions during the time the intervention was in place.

Conclusions

Shielding was a largely untested strategy, aiming to mitigate risk. The model of its rationale, components, and outcomes (intended and unintended) will inform evaluation of the impact of shielding and help us to understand its effect and limitations.

For peer review only - http://bmjopen.bmj.com/site/about/guidelines.xhtml

Strengths and limitations

- This paper presents the first description of the rationale for shielding which was an internationally unique and untested public health intervention implemented in the UK during the COVID 19 pandemic
- Our paper combines formal documentation on the shielding progamme in the UK with interviews with those with those involved in creating and implementing it, so allowing for an exploration of how the rapidly implemented policy was operationalised on the ground
- This logic model provides the first step in undertaking the EVITE Immunity study, a population-scale national assessment of effects of shielding on COVID-19 infection rate, mortality, serious illness, use of NHS resources, health-related quality-of-life and behaviour.
- While we collected views from policy makers in England and Wales, the majority of interview participants were based in Wales, so their experience may not be representative of all other parts of the UK.
- Developing this logic model within EVITE Immunity study has involved people with direct experience of shielding from the outset, with public contributors represented across all aspects of research development and implementation, reflecting strong views that evidence about effects of shielding is needed.

Main text: 3832 words

For peer review only

For peer review only - http://bmjopen.bmj.com/site/about/guidelines.xhtml

Background

As an early response to the COVID-19 pandemic, the four UK nations introduced a policy of 'shielding' for clinically extremely vulnerable (CEV) people. Those identified as being at the highest risk from COVID-19 infection, due to pre-existing conditions such as lung disease or current immunosuppressant medications, were strongly advised to strictly self-isolate, not leaving the home unless it was vital. The policy was the subject of rapid development and implementation. It was first discussed by SAGE (UK's Scientific Advisory Group for Emergencies) on 13th March 2020, and put into place within ten days. To support the shielding policy, a programme of practical and financial support was made available by a range of statutory, commercial and third sector partners.

The shielding intervention was in place for a total of 10 months over two periods, before being suspended in the spring of 2021. It eventually included over 4 million people across the UK [1].

Shielding was introduced as a novel precautionary response to an unprecedented situation, with no underpinning empirical evidence about its effectiveness at reducing SARS-CoV-2 infections, serious illness or deaths.

We undertook an evaluation of shielding in Wales (EVITE Immunity), where records for the 130,000 people who were identified for shielding are already anonymously linked with other integrated data sources, utilising the MRC funded ConCOV project in the Secure Anonymised Information Linkage (SAIL) Databank [2]. Initial findings show that people were more likely to have been identified for inclusion in the shielding intervention with increasing age, frailty and residence in deprived areas; and that reported infection rate was higher in the shielded cohort than the non-shielded general population, though testing rates were higher and infection rates amongst those not tested in each cohort were unknown [3]. We will also report how shielding affected deaths, health care utilisation, immunity status, NHS costs, and quality-of-life, and how people complied with guidance [4].

In line with the latest MRC guidelines on evaluating complex interventions [5], the first stage of the EVITE Immunity study aimed to develop a programme theory to explain the intentions of the policy, making explicit all components of the intervention (defined here as

For peer review only - http://bmjopen.bmj.com/site/about/guidelines.xhtml

being the shielding policy plus support programme), and representing these in a logic model, presented in this paper. Logic models can have a particular value in helping to articulate causality in the evaluation of public health interventions [6]. We will use this logic model to guide the analysis and interpretation of evaluation findings.

Methods

Our study was designed as case study research. Based on published information, we prepared a draft logic model describing the components of the intervention, the mechanisms by which it was assumed to work, outcomes, intended impact, risks, and relevant contextual factors. We conducted individual interviews (n=3) and group interviews (n=4) online with a total of 13 key stakeholders: senior policymakers and clinicians from Public Health and Chief Medical Officers' teams, and representatives from local government and the voluntary and community sector, in Wales (n=12) and England (n=1). Respondents were recruited to provide a range of relevant perspectives. Interviews were conducted by experienced qualitative researchers from Swansea University. In advance of the interview, we shared an information sheet on the study with participants and they completed written consent forms.

We used a semi-structured interview schedule [Appendix] to explore the rationale for shielding, steps undertaken to create and implement the intervention, and individual/organisational roles. We showed participants the draft logic model and invited comments and discussion. Data collection took place between March and May 2021.

We recorded and transcribed interviews, with participants' consent. We analysed the data using a framework approach to thematic analysis, incorporating a tabular data summary of cases/codes and data extracts [7], and refined the logic model into a final version. Each transcript was reviewed and coded by two members of the study team; findings and implications were discussed by the whole study team, including public participants.

Patient and public involvement

For peer review only - http://bmjopen.bmj.com/site/about/guidelines.xhtml

For peer review only

People affected by the shielding policy have been directly involved throughout development of the research design [8-10]. Two were co-applicants on the funding proposal and are members of the Research Management Group overseeing study implementation (LG, LD). They work with six more public contributors via a Patient Advisory Panel. An independent Study Steering Committee includes two further public contributors. Our public contributors and some academic co-applicants were personally, directly or indirectly, affected by the implementation of the shielding policy.

Results

The final version of the logic model describing the shielding intervention is shown in Figure 1. This incorporates some changes made following analysis of the interviews, including reduced deaths among the shielding population being highlighted as the primary intended impact, while the proximate outcome of 'avoided infection' was changed to 'reduced infection'. Small additions were made to inputs, context and unintended impacts. Below, we present and discuss the key aspects of the logic model and report the experience and reflections of the stakeholders we interviewed.

[Figure 1 Logic Model describing the shielding intervention]

Inputs: components of the intervention

The intervention was complex. There were three core processes relevant to all people advised to shield: the Chief Medical Officers of the four nations reaching agreement on the clinical criteria for inclusion on the CEV list; identification of people to be added to the list; and communication with those identified.

Identifying and communicating with CEV people took place in phases, with batches of individuals being added to the list over the time the shielding programme was 'live' and much smaller numbers being removed. Across the UK, CEV people were identified through searches of centralised databases (which produced the majority of names for the list in Wales), and through primary and secondary care records, in conjunction with doctors' clinical judgement [11]. This mixed approach was described as '*build the list nationally first and then ask GPs to review*' (Participant 2 Policy Maker). Some GPs and practice managers

For peer review only - http://bmjopen.bmj.com/site/about/guidelines.xhtml
For peer review only

responded to people requesting to be removed from, or added to, the list. As one respondent noted, this was more significant than a mere administrative process:

> '*The GP had the authority to put people on and off, but a lot of the GPs hadn't realised… what the implication of that was for the individual about going back to work, back to school, or actually getting access to the food box or not.*' (Participant 9 Local government)

The development of the QCovid risk prediction tool, which identified clinical and demographic risk factors for COVID-19-related hospitalisations and deaths [12 ,13] led to substantial numbers being added to CEV lists in the summer of 2020.

The 'binary' nature of inclusion on the list was seen by one respondent as problematical:

> *'It didn't reflect the spectrum of risk. So you were selecting a group of people for really quite an austere set of advisory statements, but actually nothing for the rest.' (Participant 1 Policy Maker*)

Letters were sent to CEV people strongly advising them to shield from 23rd March 2020, initially for 12 weeks [14]. Respondents reported considerable thought going into the exact wording of the letters, with an emphasis on them giving advice, not instruction:

> *'We were telling people they could rather than telling people they must. ….It was strong advice, but it was set in advisory terms.' (Participant 7 Policy maker)*

Where telephone numbers were available, reinforcement of the advice was sent by text message.

The other aspect of the intervention was the support programme, a range of components experienced by some CEV people as required. These included eligibility (achieved through a change in legislation) for Statutory Sick Pay for CEV people, even if they were not ill, to allow them to take time off work if their job could not be done at home. For those who needed them, the UK government contracted with commercial providers to deliver weekly food parcels. CEV people could also access delivery of medications and were given priority by supermarkets for food delivery.

Other forms of support were available through local government and partner organisations, and CEV people were invited to register to access this. Welsh local government respondents

For peer review only - http://bmjopen.bmj.com/site/about/guidelines.xhtml

described taking a proactive approach to contacting CEV people who might be in need of help, through welfare telephone calls:

> *'trying to keep [CEV people] linked into their society, their communities. So it was very much a kind of social response to it…Most of the people doing the calling were our librarians.'* (Participant 10 Local government)

Although changes to data sharing rules allowed lists of CEV people to be supplied to local government, significant challenges were reported with using the shared data:

> *'It was incomplete. It wasn't accessible…It wasn't transferable. It didn't match any of the other datasets. We couldn't identify individuals through it. I think the first lot didn't even have people's names. It just had their NHS number.'* (Participant 11 Local government)

Voluntary and community sector (VCS) organisations often worked in co-ordination with local and central government, running telephone helplines offering advice and emotional support for isolated people:

> *'We switched from a Monday to Friday, nine to five operation, to a seven day a week operation. …We were even doing things like contacting very local suppliers, and shops, to see if we could get deliveries put in place for people.*' (Participant 3 Voluntary and community sector)

Predicted mechanisms of change and outcome

The predicted mechanism of change was that people would isolate themselves, with the outcome of avoiding infection. Some respondents expressed anxiety about the loss of liberty this represented, even though '*it was really quite an honourable aim'* (Participant 1 Policy Maker). Initially, shielding people were advised to avoid all contact with others, even within the home. Respondents were aware that this was problematical:

> '*Everybody realised that wasn't realistic for ninety per cent of people, who don't live in houses with west wings and east wings. And so… it then became a household isolation, which was even worse.' (Participant 1 Policy Maker)*

For peer review only - http://bmjopen.bmj.com/site/about/guidelines.xhtml

Equally, some of those shielding would require care at home, with care workers a potential source of infection:

> *'A number of these people were vulnerable, they had comorbidities, they required support to manage that, and therefore contact would occur'. (Participant 5, Policy Maker)*

Respondents were aware that adherence to shielding advice would vary between individuals, and also over time, but felt that overall risk would still be reduced:

> *'It's not an all or nothing, it's not if you break it once, you've broken the law, like with the legislative regulations.' (Participant 2 Policy Maker)*

Intended impact

The primary intended impact was a reduction in mortality among CEV people. This was emphasised strongly by most respondents.

> *'The idea is that if we isolate them they're less likely to get it, because if they get it they're probably going to be really, really poorly and die.' (Participant 5, Policy Maker)*

Some respondents described a broader range of beneficial impacts, including limiting the burden on the NHS, engendering of community spirit, and more social and voluntary support for isolated vulnerable people.

> *'It's about protecting the NHS, because that's in all our interests, isn't it, but actually on a local level, it was very much about supporting our communities.' (Participant 10, Local government)*

Risks/unintended impact

All participants described potential risks or unintended impact of shielding, and in particular the impact which it might have on mental health, through isolation and anxiety:

> *'Some people who are shielding are still able to be effectively engaged somehow with society. They're working from home or whatever. But others have suffered, probably*

For peer review only - http://bmjopen.bmj.com/site/about/guidelines.xhtml

a lot…It created a whole lot of knock-on anxiety and everybody who was related in any way with the shielded person [was] put in a state of heightened awareness and heightened anxiety.' (Participant 1 Policy Maker)

Concerns about the long-term mental health impact were reported as a factor in the decision to pause shielding advice in July 2020:

'The effect on mental health started to outweigh the benefit once the prevalence was low enough.' (Participant 2 Policy Maker)

In addition, concerns were widely expressed about the impact of shielding on physical health, including muscle wastage:

'The debilitation from not leaving the house …this was not just that you were telling ninety- year-olds and eighty-year-olds, you were telling this to often quite fit young people who just had another condition to stay in the house.' (Participant 4, Policy Maker)

Other unintended impacts included that on the workforce, as CEV people whose jobs could not be done from home were no longer available to work.

Contextual factors

The shielding policy was based on the assumption that the CEV group would have enhanced protection over that of the general population. Almost as soon as shielding started, a lockdown was introduced for the wider population, imposing legally mandated restrictions on spending time outside the home except for certain exemptions. The national lockdown also brought certain whole-population initiatives, such as the 'furlough scheme' to subsidise wages for staff on temporary leave.

Respondents reflected that although lockdown was likely to have slowed the spread of the virus in the wider population, continuing steady rates of infection meant that shielding needed to continue to run in parallel:

'It went on an awful lot longer than was envisaged in the first instance.' (Participant 4 Policy Maker)

For peer review only - http://bmjopen.bmj.com/site/about/guidelines.xhtml

Since the shielding policy was developed in the context of a crisis, it - along with the associated shielding programme - was devised and implemented at an unusually fast rate, and participants spoke of a clear shared purpose:

> *'We all had to make decisions differently and quickly, and we all had to use a bit more common sense than the traditional waiting for somebody else to come up with a strategy.' (Participant 8 Local government)*

Local government respondents reported that there was a blurring around the edges of the population receiving their input, as they added people they knew to be vulnerable to their lists for this type of support, in addition to CEV people on the shielding list.

> *'Our proactive calling quickly extended to older people who were not necessarily shielding, but we felt they were also vulnerable – a number of them were… in effect shielding themselves.' (Participant 10 Local government)*

In addition to the formal social support available from local authorities and third sector organisations, many CEV people were likely to have been able to access a wide range of other resources within the community, which enabled shielding to happen, especially informal support from family and friends. However, some would have lacked informal support from family, friends, and neighbours and may have become increasingly isolated.

Discussion

Principal findings

Our logic model provides a visual presentation of our understanding of the programme theory underlying the shielding intervention introduced across the UK to protect the most vulnerable people doing the early phases of the COVID-19 pandemic. It captures the key components of the shielding intervention (policy plus support programme), and identifies the mechanism by which it might make a difference, potential impacts, both unintended and intended, and key contextual factors. The logic model will underpin our evaluation of the impact of the shielding intervention, and we will continue to review it as the evaluation is finalised, in order to provide a structure to evaluating an intervention which to an extent was fluid and extemporised.

For peer review only - http://bmjopen.bmj.com/site/about/guidelines.xhtml

Our interviews with the key stakeholders add three main areas of insight: into the iterative and fluid process of developing and implementing shielding; into the range of activities which local government and the third sector introduced to support shielding people, outside the formal bounds of the programme; and into the feelings of many of those involved in the process, who revealed uncertainty about not only the logic but also the justice of shielding. The shielding policy, like many other aspects of the UK government's response to SARS-CoV-2, was introduced at pace. This left those involved in implementing the policy and delivering the shielding programme to work out the details at speed, once the decision had been made. The policy implementation and support programme were designed and iteratively refined by the many different parties collaborating on the work across the four nations of the UK – not just SAGE, civil servants and clinicians, but also those involved in providing services at local and community level. In the delivery of the shielding support programme, there was some blurring, as the formal shielding policy ended up being delivered alongside support interventions for those regarded as vulnerable for social rather than clinical reasons. This does not undermine the programme theory, but instead is integral to it.

The nature of implementation as a process over time could also be observed. Although the basic principle of shielding remained the same throughout the time period of the intervention, the details evolved significantly – in terms of the nature of the advice, the definition of who should be on the shielding list, and in turn, the numbers of those included. As the pandemic continued, the evolution of the innovative attempts to deal with its impact were obvious, with new ways of working emerging between national government, local government and the voluntary and community sector.

There was some ambiguity about who was doing the shielding: the use of the term 'Shielded Patient List' in guidance from the Department of Health and Social Care [15] implies that those at risk were being shielded by the state; yet the advisory nature of the guidance suggests that people were being asked to actively shield themselves – shielding rather than being shielded.

For peer review only - http://bmjopen.bmj.com/site/about/guidelines.xhtml

Context of other literature

The changes in the intervention over time noted in our study reflect the evolving nature of the shielding list, and the slippage between guidance and advice in public discourse, has been tracked in detail by Herrick [16]. Emerging evidence suggests that, despite the shielding intervention, there were still high rates of infection, hospitalisation and mortality in the shielding group [3,17] casting doubt on the mechanism proposed. This may in part be due to the impracticality of truly isolating people, particularly those who were in contact with clinical care providers and carers due to their vulnerability. Modelling has suggested that under an 'imperfect' but realistic shielding strategy, in which contacts for those shielding were reduced by 80%, would still allow high rates of infection of high risk individuals, with deaths estimated at 150% -300% higher than under an implausible 'perfect' shielding model in which contacts were reduced to zero [18]. High rates of nosocomial COVID-19 infection have been identified, likely to disproportionately affect CEV people [19-21].

Our findings confirm previous studies which have identified the crucial role of local government and VCS organisations in supporting the implementation and operationalisation of the shielding policy [22], and previous work discussing local variation in how the CEV list was created and the proportion and make-up of population on it [1], in particular associated with the addition of people to the list by local clinicians [23]. Communication with CEV people to inform them of the support available has been reported elsewhere as missing thousands of people, as records were incomplete [23]. A rapid evaluation in Scotland concluded that while the principle of shielding was valid, the intervention should not be repeated in exactly the same format [24].

The concerns expressed in our interviews about potential negative impacts of shielding reflect suggestions from other studies that people advised to shield may have experienced increased anxiety and mental ill-health and struggled to access routine healthcare [1 ,25], and there may have been an additional strain on unpaid carers who were left without their usual support [26].

For peer review only - http://bmjopen.bmj.com/site/about/guidelines.xhtml

Strengths and limitations

This is the first study to develop a logic model examining all components of the UK COVID-19 shielding policy and programme, and to report the perspective of those involved in operationalising it.

In this phase of the EVITE Immunity evaluation, we did not record the perspective of CEV people themselves, who will have adhered to a greater or lesser extent, with adherence perhaps changing over time [11]; later phases of EVITE Immunity will explore the experience of this group.

Implications

The UK's response to the pandemic of advising shielding for the most vulnerable was an unusual one, paralleled most closely in Europe by Ireland and by Sweden, where a policy of shielding people aged over 70 contrasted with the general population lock downs of its Nordic neighbours [27]. Although initial modelling in the UK explored a similar community level, age-based approach to shielding [28 ,29], the adoption instead of an approach based on identifying and targeting individual clinical vulnerability was substantially more complex, and perhaps was regarded as more acceptable to the public. The emphasis in the UK on a mitigation approach – reducing the peak of infection while protecting the vulnerable – was soon overtaken by the imposition of general population lockdowns, but the two policy approaches continued to run in parallel, at least initially. Modelling early in the pandemic had identified a potential trade-off between increasing protection of the vulnerable and relaxing restrictions on the non-vulnerable [11]. However, the overlapping lockdown and shielding restrictions in the UK, together with the 'leakage' of shielding through necessary personal contacts, along with rates of full compliance reported as down to less than two thirds of those on the list by early summer 2020 [30] make it hard to measure how this has played out.

Over time, there has been an evolution of risk within the shielded population resulting from subsequent waves of coronavirus infection, along with the vaccination programme. Shielding has now formally ended in both England and Wales. The most recent government guidance to previously shielding people in England reassured that, with protection from the vaccine, they would no longer be at substantially greater risk than the rest of the population

For peer review only

[31], though new mutations challenge the efficacy of existing vaccines. A now much better defined core high risk subset remains, consisting of those unable to respond to vaccination appropriately [32].

In terms of both infection rates and mortality in the UK, the pandemic has been identified as having an unequal impact on the population, reflecting health inequalities ultimately rooted in social inequalities [32]. The shielding policy may have exacerbated these, as those living in more crowded accommodation would have found it more challenging to maintain isolation from others.

Conclusion

The shielding intervention was introduced to save lives by protecting the most vulnerable to SARS-COV-2 infection. The shielding programme of support was introduced particularly rapidly and involved novel collaborations between various agencies. Components varied slightly but were broadly similar across the UK. It was a hitherto largely untested strategy based on 'common sense' risk mitigation rather than evidence based interventions.

Naturally, this large scale initiative created challenges both for those attempting to implement the policy and for those meant to benefit from it. Our logic model allows us to understand the different impacts (intended and unintended) of the shielding programme on organisations and populations, and spells out its rationale, components, and mechanisms. Developing the logic model with input from key stakeholders has given additional insight to help us understand the causal links which will inform our evaluation of the impact of shielding and help us to understand its effect and limitations.

Ethics:

The EVITE Immunity study has received approval from the Newcastle North Tyneside 2 Research Ethics Committee (IRAS 295050).

Funding statement:

This work is supported by the National Core Studies Immunity (NCSi4P) Programme.

For peer review only - http://bmjopen.bmj.com/site/about/guidelines.xhtml

Transparency statement:

Alison Porter is the manuscript's guarantor and affirms that the manuscript is an honest, accurate, and transparent account of the study being reported; that no important aspects of the study have been omitted; and that any discrepancies from the study as originally planned (and, if relevant, registered) have been explained.

Competing interests:

Ronan Lyons, Stephen Jolles, Ann John and Adrian Edwards are members of the Welsh Government COVID-19 Technical Advisory Group. Ann John is also co-Chair of the Scientific Pandemic Insights Group on Behaviours (SPI-B) which is a subgroup of the Scientific Advisory Group for Emergencies (SAGE) advising the UK government. Stephen Jolles is also a member of the Welsh Government Testing TAG and Cardiff University COVID Strategic Advisory Board (SAB). No other authors have competing interests.

For peer review only - http://bmjopen.bmj.com/site/about/guidelines.xhtml

References

1. Hodgson K, Butler JE, Davies A, et al. Briefing: Assessing the impact of COVID-19 on the clinically extremely vulnerable population. Health Foundation. 2021
2. Lyons J, Akbari A, Torabi F, et al. Understanding and responding to COVID-19 in Wales: protocol for a privacy-protecting data platform for enhanced epidemiology and evaluation of interventions. *BMJ Open* 2020;10(10):e043010.
3. Snooks H, Watkins A, Lyons J et al. Did the UK's public health Shielding policy protect the clinically extremely vulnerable during the COVID-19 pandemic in Wales? Results of EVITE Immunity, a linked data retrospective study. *Public Health* 2023 in press https://doi.org/10.1016/j.puhe.2023.02.008
4. Evans BA, Akbari A, Bailey R, et al. Evaluation of the shielding initiative in Wales (EVITE Immunity): protocol for a quasiexperimental study. *BMJ Open* 2022;12(9):e059813.
5. Skivington K, Matthews L, Simpson SA, et al. A new framework for developing and evaluating complex interventions: update of Medical Research Council guidance. *BMJ* 2021;374:n2061. doi: 10.1136/bmj.n2061 [published Online First: 20210930]
6. Langley T, Gillespie D, Lewis S, et al. Developing logic models to inform public health policy outcome evaluation: an example from tobacco control. *Journal of Public Health* 2021;43(3):639-46.
7. Ritchie J, Spencer L. Qualitative data analysis for applied policy research. Analyzing qualitative data: Routledge 2002:187-208.
8. National Institute for Health Research. UK Standards for Public Involvement., 2019.
9. Staniszewska S, Brett J, Mockford C, et al. The GRIPP checklist: strengthening the quality of patient and public involvement reporting in research. *Int J Technol Assess Health Care* 2011;27(4):391-9. doi: 10.1017/S0266462311000481
10. Evans BA, Gallanders J, Griffiths L, et al. Public involvement and engagement in primary and emergency care research: the story from PRIME Centre Wales. *International Journal of Population Data Science* 2020;5(3)
11. Davies G. Protecting and supporting the clinically extremely vulnerable during lockdown. London: National Audit Office, Department of Health and Social Care,, 2021.
12. Clift AK, Coupland CAC, Keogh RH, et al. Living risk prediction algorithm (QCOVID) for risk of hospital admission and mortality from coronavirus 19 in adults: national derivation and validation cohort study. *BMJ* 2020;371:m3731. doi: 10.1136/bmj.m3731 [published Online First: 20201020]
13. Lyons J, Nafilyan V, Akbari A, et al. Validating the QCOVID risk prediction algorithm for risk of mortality from COVID-19 in the adult population in Wales, UK. *Int J Popul Data Sci* 2020;5(4):1697. doi: 10.23889/ijpds.v5i4.1697 [published Online First: 20220215]
14. Welsh Government. Guidance on protecting people defined on medical grounds as clinically extremely vulnerable from coronavirus (COVID-19) – previously known as 'shielding'. Information for protecting people defined on medical grounds as extremely vulnerable from COVID-19., 2022.
15. Department of Health and Social Care. Guidance on protecting people who are clinically extremely vulnerable from COVID-19 In:England., 2021.
16. Herrick C. 'We thank you for your sacrifce': Clinical vulnerability, shielding and biosociality in the UK's Covid-19 response. Biosocieties Jan 22;1-23. doi: 10.1057/s41292-021-00266-0
17. Jani BD, Ho FK, Lowe DJ, et al. Comparison of COVID-19 outcomes among shielded and non-shielded populations. *Scientific Reports* 2021;11(1):15278. doi: 10.1038/s41598-021-94630-6
18. Smith CA, Yates CA, Ashby B. Critical weaknesses in shielding strategies for COVID-19. PLOS Glob Public Health, 2022 2(4): e0000298.

For peer review only

For peer review only - http://bmjopen.bmj.com/site/about/guidelines.xhtml

19. Ponsford MJ, Jefferies R, Davies C, et al. Burden of nosocomial COVID-19 in Wales: results from a multicentre retrospective observational study of 2508 hospitalised adults. *Thorax* 2021;76(12):1246-49.
20. Ponsford MJ, Ward TJ, Stoneham SM, et al. A systematic review and meta-analysis of inpatient mortality associated with nosocomial and community COVID-19 exposes the vulnerability of immunosuppressed adults. *Frontiers in immunology* 2021;12:744696.
21. McKeigue PM, McAllister DA, Caldwell D, et al. Relation of severe COVID-19 in Scotland to transmission-related factors and risk conditions eligible for shielding support: REACT-SCOT case-control study. *BMC medicine* 2021;19(1):1-13.
22. Bevan Foundation. Lessons from lockdown: the experience of shielding. 2020 [Available from: https://www.bevanfoundation.org/resources/experience-of-shielding/.
23. House of Commons Committee of Public Accounts. COVID-19: supporting the vulnerable during lockdown. Fifty-third report of Session 2019-21. HC938. 2021
24. Public Health Scotland. Covid-19 Shielding Programme (Scotland) Rapid Evaluation: Full Report. Edinburgh: Public Health Scotland, 2021.
25. Fisher A, Roberts A, McKinlay AR, et al. The impact of the COVID-19 pandemic on mental health and well-being of people living with a long-term physical health condition: a qualitative study. *BMC Public Health* 2021;21(1):1-12.
26. Lorenz-Dant K, Comas-Herrera A. The impacts of COVID-19 on unpaid carers of adults with long-term care needs and measures to address these impacts: A rapid review of evidence up to November 2020. *Journal of Long-Term Care* 2022;2021:124-53.
26. Davies NG, Kucharski AJ, Eggo RM, et al. Effects of non-pharmaceutical interventions on COVID-19 cases, deaths, and demand for hospital services in the UK: a modelling study. *The Lancet Public Health* 2020;5(7):e375-e85.
28. Ferguson NM, Laydon D, Nedjati-Gilani G, et al. Impact of non-pharmaceutical interventions (NPIs) to reduce COVID-19 mortality and healthcare demand. 2020
29. Van Bunnik BA, Morgan AL, Bessell PR, et al. Segmentation and shielding of the most vulnerable members of the population as elements of an exit strategy from COVID-19 lockdown. *Philosophical Transactions of the Royal Society B* 2021;376(1829):20200275.
30. Department of Health and Social Care. Guidance for people previously considered clinically extremely vulnerable from COVID-19., 2022.
31. Ponsford MJ, Evans K, Carne EM, et al. COVID-19 Vaccine Uptake and Efficacy in a National Immunodeficiency Cohort. *Journal of Clinical Immunology* 2022;42(4):728-31. doi: 10.1007/s10875-022-01223-7
32. Jung A-S, Haldane V, Neill R, et al. National responses to covid-19: drivers, complexities, and uncertainties in the first year of the pandemic. *BMJ* 2021;375

For peer review only - http://bmjopen.bmj.com/site/about/guidelines.xhtml

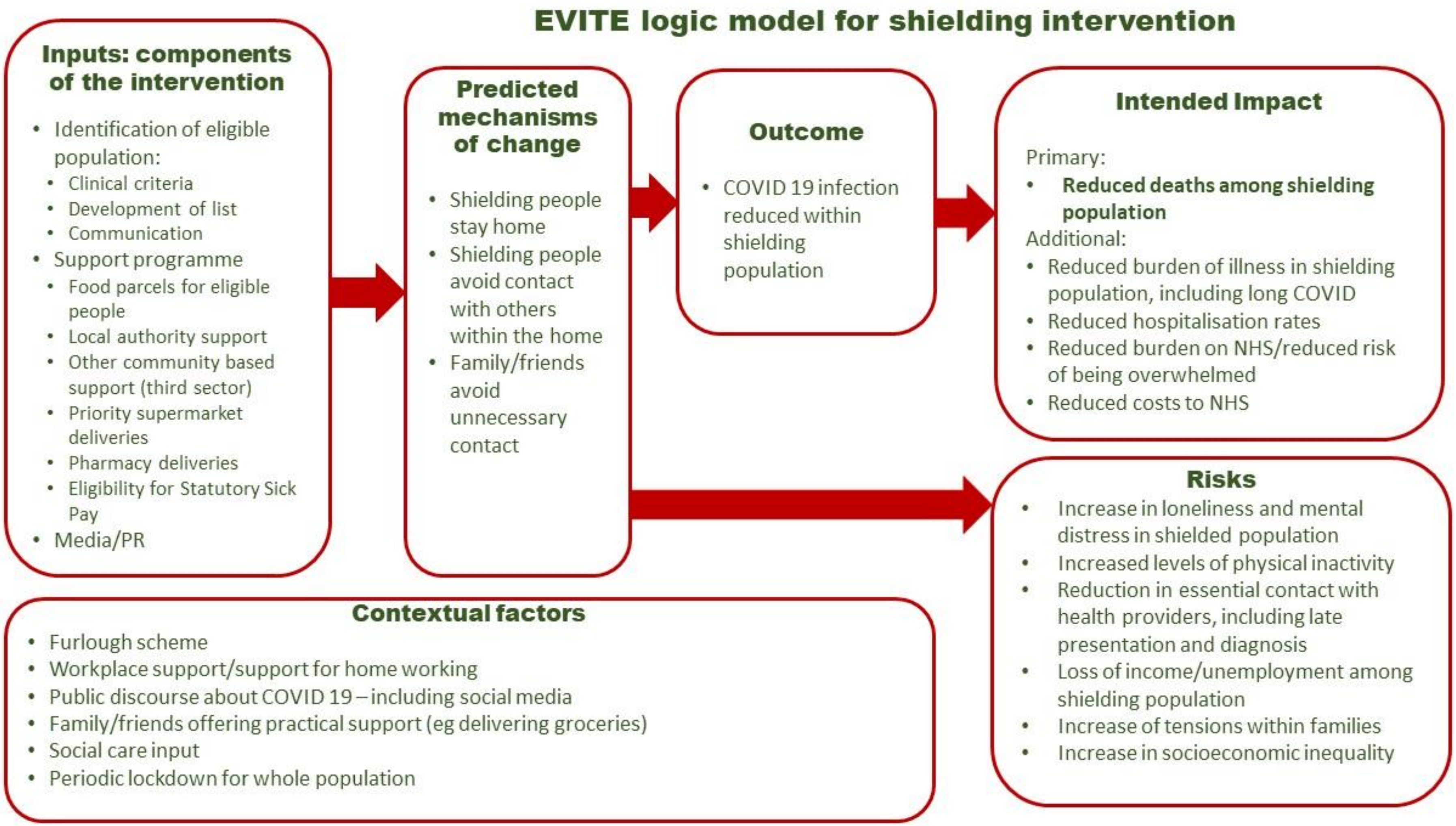
EVITE logic model for shielding intervention
Inputs: components of the intervention
Identification of eligible population:
Clinical criteria
Development of list
Communication
Support programme
Food parcels for eligible people
Local authority support
Other community based support (third sector)
Priority supermarket deliveries
Pharmacy deliveries
Eligibility for Statutory Sick Pay
Media/PR
Predicted mechanisms of change
Shielding people stay home
Shielding people avoid contact with others within the home
Family/friends avoid unnecessary contact
Outcome
COVID 19 infection reduced within shielding population
Intended Impact
Primary:
Reduced deaths among shielding population
Additional:
Reduced burden of illness in shielding population, including long COVID
Reduced hospitalisation rates
Reduced burden on NHS/reduced risk of being overwhelmed
Reduced costs to NHS
Risks
Increase in loneliness and mental distress in shielded population
Increased levels of physical inactivity
Reduction in essential contact with health providers, including late presentation and diagnosis
Loss of income/unemployment among shielding population
Increase of tensions within families
Increase in socioeconomic inequality
Contextual factors
Furlough scheme
Workplace support/support for home working
Public discourse about COVID 19 – including social media
Family/friends offering practical support (eg delivering groceries)
Social care input
Periodic lockdown for whole population

Appendix **EVITE Immunity Phase 1 – WP1 QUESTIONS FOR STAKEHOLDER CONSULTATION**

This consultation is about understanding the rationale for the shielding programme in Wales for patients clinically extremely vulnerable to COVID-19 infection.

1. What was the objective of the shielding programme?
    - To reduce mortality among vulnerable people
    - To limit pressure on the NHS
    - To reduce infection rates/morbidity
    - Were there multiple objectives? If so, was one more important that the others?
    - Did the objectives change over time?

2. How was the shielding programme designed to work?
    - Change in behaviour of people at risk (staying home/avoiding contact)
    - Change in behaviour of other household members (staying home/avoiding contact)
    - Adaptations made by employers (allowing people to work from home, furloughing staff)
    - Increasing awareness of those clinically vulnerable aka the shielding population
    - Increasing awareness of risk among the wider public
    - Was there any change over time in how the programme was designed to work
3. What was needed to make it work?
    - Identifying people at risk – datasets, risk algorithms, clinical judgement
    - Information/communication with people on the list (letter vs text mechanism and accessibility of these)
    - Support/advice from medical staff (GPs or consultants managing patients' conditions)
    - Support/advice from carers of those identified as clinically vulnerable
    - Food parcels
    - Benefits/compensation for people unable to work
    - Have all these been in place? Has anything changed over time?

4. How was the shielding programme developed?
    - Where did the idea come from?
    - Who was involved in developing the programme?
    - Who made the decisions?
    - What contact was there with partners across the UK?
    - How did the shielding programme in Wales relate to England?
    - as there consensus among all involved about the objectives of the programme and what form it should take?

5. How has the shielding programme been reviewed/monitored?
    - What information has been gathered?
    - What has changed as a result?

For peer review only - http://bmjopen.bmj.com/site/about/guidelines.xhtml

1

Standards for Reporting Qualitative Research (SRQR)*

http://www.equator-network.org/reporting-guidelines/srqr/

Title and abstract

	Page/line no(s).
**Title** - Concise description of the nature and topic of the study Identifying the study as qualitative or indicating the approach (e.g., ethnography, grounded theory) or data collection methods (e.g., interview, focus group) is recommended	P1
**Abstract** - Summary of key elements of the study using the abstract format of the intended publication; typically includes background, purpose, methods, results, and conclusions	P2

Introduction

**Problem formulation** - Description and significance of the problem/phenomenon studied; review of relevant theory and empirical work; problem statement	P4
**Purpose or research question** - Purpose of the study and specific objectives or questions	P4

Methods

**Qualitative approach and research paradigm** - Qualitative approach (e.g., ethnography, grounded theory, case study, phenomenology, narrative research) and guiding theory if appropriate; identifying the research paradigm (e.g., postpositivist, constructivist/ interpretivist) is also recommended; rationale**	P5
**Researcher characteristics and reflexivity** - Researchers' characteristics that may influence the research, including personal attributes, qualifications/experience, relationship with participants, assumptions, and/or presuppositions; potential or actual interaction between researchers' characteristics and the research questions, approach, methods, results, and/or transferability	P5
**Context** - Setting/site and salient contextual factors; rationale**	P5
**Sampling strategy** - How and why research participants, documents, or events were selected; criteria for deciding when no further sampling was necessary (e.g., sampling saturation); rationale**	P5
**Ethical issues pertaining to human subjects** - Documentation of approval by an appropriate ethics review board and participant consent, or explanation for lack thereof; other confidentiality and data security issues	P15
**Data collection methods** - Types of data collected; details of data collection procedures including (as appropriate) start and stop dates of data collection and analysis, iterative process, triangulation of sources/methods, and modification of procedures in response to evolving study findings; rationale**	P5

For peer review only - http://bmjopen.bmj.com/site/about/guidelines.xhtml

For peer review only

**Data collection instruments and technologies** - Description of instruments (e.g., interview guides, questionnaires) and devices (e.g., audio recorders) used for data collection; if/how the instrument(s) changed over the course of the study	P5
**Units of study** - Number and relevant characteristics of participants, documents, or events included in the study; level of participation (could be reported in results)	P5
**Data processing** - Methods for processing data prior to and during analysis, including transcription, data entry, data management and security, verification of data integrity, data coding, and anonymization/de-identification of excerpts	P5
**Data analysis** - Process by which inferences, themes, etc., were identified and developed, including the researchers involved in data analysis; usually references a specific paradigm or approach; rationale**	P5
**Techniques to enhance trustworthiness** - Techniques to enhance trustworthiness and credibility of data analysis (e.g., member checking, audit trail, triangulation); rationale**	P5

Results/findings

**Synthesis and interpretation** - Main findings (e.g., interpretations, inferences, and themes); might include development of a theory or model, or integration with prior research or theory	Pp6-11
**Links to empirical data** - Evidence (e.g., quotes, field notes, text excerpts, photographs) to substantiate analytic findings	Pp6-11

Discussion

**Integration with prior work, implications, transferability, and contribution(s) to the field -** Short summary of main findings; explanation of how findings and conclusions connect to, support, elaborate on, or challenge conclusions of earlier scholarship; discussion of scope of application/generalizability; identification of unique contribution(s) to scholarship in a discipline or field	Pp11-13
**Limitations** - Trustworthiness and limitations of findings	P14

Other

**Conflicts of interest** - Potential sources of influence or perceived influence on study conduct and conclusions; how these were managed	P16
**Funding** - Sources of funding and other support; role of funders in data collection, interpretation, and reporting	P15

*The authors created the SRQR by searching the literature to identify guidelines, reporting standards, and critical appraisal criteria for qualitative research; reviewing the reference lists of retrieved sources; and contacting experts to gain feedback. The SRQR aims to improve the transparency of all aspects of qualitative research by providing clear standards for reporting qualitative research.

For peer review only - http://bmjopen.bmj.com/site/about/guidelines.xhtml

**The rationale should briefly discuss the justification for choosing that theory, approach, method, or technique rather than other options available, the assumptions and limitations implicit in those choices, and how those choices influence study conclusions and transferability. As appropriate, the rationale for several items might be discussed together.

Reference:

O'Brien BC, Harris IB, Beckman TJ, Reed DA, Cook DA. **Standards for reporting qualitative research: a synthesis of recommendations.** *Academic Medicine*, Vol. 89, No. 9 / Sept 2014 DOI: 10.1097/ACM.0000000000000388

For peer review only

For peer review only - http://bmjopen.bmj.com/site/about/guidelines.xhtml

BMJ Open

Rationale for the shielding policy for clinically vulnerable people in the UK during the COVID-19 pandemic: a qualitative study

Journal:	*BMJ Open*
Manuscript ID	bmjopen-2023-073464.R1
Article Type:	Original research
Date Submitted by the Author:	18-May-2023
Complete List of Authors:	Porter, Alison; Swansea University, Swansea University Medical School<br>Akbari, Ashley; Swansea University Medical School,<br>Carson-Stevens, A; Cardiff University<br>Dale, Jeremy; University of Warwick, Warwick Medical School<br>Dixon, Lucy; Public contributor<br>Edwards, Adrian; Cardiff University,<br>Griffiths, Lesley; Public Contributor<br>John, Ann; Swansea University, Swansea University Medical School<br>Jolles, Stephen; Cardiff University,<br>Kingston, Mark; Swansea University, Medicine<br>Lyons, Ronan; University of Wales Swansea, Swansea Clinical School<br>Morgan, Jennifer; NHS Wales, Delivery Unit<br>Sewell, Bernadette; Swansea University, College of Human and Health Sciences<br>Williams, Victoria; Swansea University, Swansea University Medical School<br>Snooks, Helen; Swansea University, Medicine
**Primary Subject Heading**:	Public health
Secondary Subject Heading:	Health services research
Keywords:	Public health < INFECTIOUS DISEASES, QUALITATIVE RESEARCH, PUBLIC HEALTH, COVID-19, Protocols & guidelines < HEALTH SERVICES ADMINISTRATION & MANAGEMENT

SCHOLARONE™
Manuscripts

I, the Submitting Author has the right to grant and does grant on behalf of all authors of the Work (as defined in the below author licence), an exclusive licence and/or a non-exclusive licence for contributions from authors who are: i) UK Crown employees; ii) where BMJ has agreed a CC-BY licence shall apply, and/or iii) in accordance with the terms applicable for US Federal Government officers or employees acting as part of their official duties; on a worldwide, perpetual, irrevocable, royalty-free basis to BMJ Publishing Group Ltd ("BMJ") its licensees and where the relevant Journal is co-owned by BMJ to the co-owners of the Journal, to publish the Work in this journal and any other BMJ products and to exploit all rights, as set out in our licence.

The Submitting Author accepts and understands that any supply made under these terms is made by BMJ to the Submitting Author unless you are acting as an employee on behalf of your employer or a postgraduate student of an affiliated institution which is paying any applicable article publishing charge ("APC") for Open Access articles. Where the Submitting Author wishes to make the Work available on an Open Access basis (and intends to pay the relevant APC), the terms of reuse of such Open Access shall be governed by a Creative Commons licence – details of these licences and which Creative Commons licence will apply to this Work are set out in our licence referred to above.

Other than as permitted in any relevant BMJ Author's Self Archiving Policies, I confirm this Work has not been accepted for publication elsewhere, is not being considered for publication elsewhere and does not duplicate material already published. I confirm all authors consent to publication of this Work and authorise the granting of this licence.

For peer review only

Title	Rationale for the shielding policy for clinically vulnerable people in the UK during the COVID-19 pandemic: a qualitative study
Authors' names	Alison Porter (0000-0002-3408-7007), Ashley Akbari, Andrew Carson-Stevens, Jeremy Dale, Lucy Dixon, Adrian Edwards, Bridie Evans, Lesley Griffiths, Ann John, Stephen Jolles, Ronan Lyons, Mark Kingston, Jennifer Morgan, Bernadette Sewell, Anthony Whiffen, Victoria Williams, Helen Snooks
Address for each author	Swansea University Medical School, ILS2, Singleton Park, Swansea SA2 8PP, UK Alison Porter Associate Professor of Health Services Research; Swansea University Medical School Ashley Akbari Senior Research Manager HDR-UK; Cardiff University UK Andrew Carson-Stevens Professor of Primary Care and Safety; Warwick University Medical School UK Jeremy Dale Professor of Primary Care; C/o Swansea University Medical School UK Lucy Dixon public representative; Cardiff University UK Adrian Edwards Professor of General Practice; Swansea University Medical School UK Bridie Evans Research Officer; C/o Swansea University Medical School UK Lesley Griffiths public representative; Swansea University Medical School UK Ann John Professor of Public Health Psychiatry; Cardiff University UK Stephen Jolles Professor of Clinical Immunology; Swansea University Medical School UK Mark Kingston Research Officer; Swansea University Medical School UK Ronan Lyons Professor of Public Health and Informatics; NHS Wales Delivery Unit UK Jennifer Morgan Operational Research and Modelling Development Manager; Swansea University Medical School UK Bernadette Sewell Health Economist; Administrative Data Research Unit, Welsh Government, UK Anthony Whiffen; Swansea University Medical School UK Victoria Williams Research Officer; Swansea University Medical School UK Helen Snooks Professor of Health Services Research.
Corresponding author	Alison Porter a.m.porter@swansea.ac.uk

Copyright: The Corresponding Author has the right to grant on behalf of all authors and does grant on behalf of all authors, an exclusive licence (or non exclusive for government employees) on a worldwide basis to the BMJ Publishing Group Ltd to permit this article (if accepted) to be published in BMJ editions and any other BMJPGL products and sublicences such use and exploit all subsidiary rights, as set out in our licence.

For peer review only - http://bmjopen.bmj.com/site/about/guidelines.xhtml

ABSTRACT

Introduction

Shielding aimed to protect those predicted to be at highest risk from COVID-19 and was uniquely implemented in the United Kingdom during the first year of the pandemic, from March 2020. As the first stage in the EVITE Immunity evaluation, we generated a logic model to describe the programme theory underlying the shielding intervention.

Design and participants

We reviewed published documentation on shielding to develop an initial draft of the logic model. We then discussed this draft during interviews with 13 key stakeholders involved in putting shielding into effect in Wales and England. Interviews were recorded, transcribed and analysed thematically to inform a final draft of the logic model.

Results

The shielding intervention was a complex one, introduced at pace by multiple agencies working together. We identified three core components: agreement on clinical criteria; development of the list of people appropriate for shielding; and communication of shielding advice. In addition, there was a support programme, available as required to shielding people, including food parcels, financial support, and social support. The predicted mechanism of change was that people would isolate themselves and so avoid infection, with the primary intended outcome being reduction in mortality in the shielding group. Unintended impacts included negative impact on mental and physical health and wellbeing. Details of the intervention varied slightly across the home nations of the UK, and were subject to minor revisions during the time the intervention was in place.

Conclusions

Shielding was a largely untested strategy, aiming to mitigate risk by placing a responsibility on individuals to protect themselves. The model of its rationale, components, and outcomes (intended and unintended) will inform evaluation of the impact of shielding and help us to understand its effect and limitations.

For peer review only - http://bmjopen.bmj.com/site/about/guidelines.xhtml

Strengths and limitations

- This paper presents the first description of the rationale for shielding which was an internationally unique and untested public health intervention implemented in the UK during the COVID 19 pandemic
- Our paper combines formal documentation on the shielding progamme in the UK with interviews with those with those involved in creating and implementing it, so allowing for an exploration of how the rapidly implemented policy was operationalised on the ground
- This logic model provides the first step in undertaking the EVITE Immunity study, a population-scale national assessment of effects of shielding on COVID-19 infection rate, mortality, serious illness, use of NHS resources, health-related quality-of-life and behaviour.
- While we collected views from policy makers in England and Wales, the majority of interview participants were based in Wales, so their experience may not be representative of all other parts of the UK.
- Developing this logic model within EVITE Immunity study has involved people with direct experience of shielding from the outset, with public contributors represented across all aspects of research development and implementation, reflecting strong views that evidence about effects of shielding is needed.

Main text: 3892 words

For peer review only - http://bmjopen.bmj.com/site/about/guidelines.xhtml

For peer review only

Background

As an early response to the COVID-19 pandemic, the four UK nations introduced a policy of 'shielding' for clinically extremely vulnerable (CEV) people. Those identified as being at the highest risk from COVID-19 infection, due to pre-existing conditions such as lung disease or current immunosuppressant medications, were strongly advised to strictly self-isolate, not leaving the home unless it was vital. The policy was the subject of rapid development and implementation. It was first discussed by SAGE (UK's Scientific Advisory Group for Emergencies) on 13th March 2020, and put into place within ten days. To support the shielding policy, a programme of practical and financial support was made available by a range of statutory, commercial and third sector partners.

The shielding intervention was in place for a total of 10 months over two periods, before being suspended in the spring of 2021. It eventually included over 4 million people across the UK [1].

Shielding was introduced as a novel precautionary response to an unprecedented situation, with no underpinning empirical evidence about its effectiveness at reducing SARS-CoV-2 infections, serious illness or deaths.

We undertook an evaluation of shielding in Wales (EVITE Immunity), where records for the 130,000 people who were identified for shielding are already anonymously linked with other integrated data sources, utilising the MRC funded ConCOV project in the Secure Anonymised Information Linkage (SAIL) Databank [2]. Initial findings show that people were more likely to have been identified for inclusion in the shielding intervention with increasing age, frailty and residence in deprived areas; and that reported infection rate was higher in the shielded cohort than the non-shielded general population, though testing rates were higher and infection rates amongst those not tested in each cohort were unknown [3]. We will also report how shielding affected deaths, health care utilisation, immunity status, NHS costs, and quality-of-life, and how people complied with guidance [4].

In line with the latest MRC guidelines on evaluating complex interventions [5], the first stage of the EVITE Immunity study aimed to develop a programme theory to explain the intentions of the policy, making explicit all components of the intervention (defined here as

For peer review only - http://bmjopen.bmj.com/site/about/guidelines.xhtml

being the shielding policy plus support programme), and representing these in a logic model, presented in this paper. Logic models can have a particular value in helping to articulate causality in the evaluation of public health interventions [6]. We will use this logic model to guide the analysis and interpretation of evaluation findings.

Methods

Our study was designed as case study research. Based on published information, we prepared a draft logic model describing the components of the intervention, the mechanisms by which it was assumed to work, outcomes, intended impact, risks, and relevant contextual factors. We conducted individual interviews (n=3) and group interviews (n=4) online with a total of 13 key stakeholders: senior policymakers and clinicians from Public Health and Chief Medical Officers' teams, and representatives from local government and the voluntary and community sector, in Wales (n=12) and England (n=1). Respondents were recruited to provide a range of relevant perspectives. Interviews were conducted by experienced qualitative researchers from Swansea University. In advance of the interview, we shared an information sheet on the study with participants and they completed written consent forms.

We used a semi-structured interview schedule [Supplementary file EVITE Immunity Rationale for shielding March 2023 Appendix Interview questions.docx (v1.0)] to explore the rationale for shielding, steps undertaken to create and implement the intervention, and individual/organisational roles. We showed participants the draft logic model and invited comments and discussion. Data collection took place between March and May 2021.

We recorded and transcribed interviews, with participants' consent. We analysed the data using a framework approach to thematic analysis, incorporating a tabular data summary of cases/codes and data extracts [7], and refined the logic model into a final version. Each transcript was reviewed and coded by two members of the study team; findings and implications were discussed by the whole study team, including public participants.

Patient and public involvement

For peer review only - http://bmjopen.bmj.com/site/about/guidelines.xhtml

People affected by the shielding policy have been directly involved throughout development of the research design [8-10]. Two were co-applicants on the funding proposal and are members of the Research Management Group overseeing study implementation (LG, LD). They work with six more public contributors via a Patient Advisory Panel. An independent Study Steering Committee includes two further public contributors. Our public contributors and some academic co-applicants were personally, directly or indirectly, affected by the implementation of the shielding policy.

Results

The final version of the logic model describing the shielding intervention is shown in Figure 1. This incorporates some changes made following analysis of the interviews, including reduced deaths among the shielding population being highlighted as the primary intended impact, while the proximate outcome of ‘avoided infection’ was changed to ‘reduced infection’. Small additions were made to inputs, context and unintended impacts. Below, we present and discuss the key aspects of the logic model and report the experience and reflections of the stakeholders we interviewed.

[Figure 1 Logic Model describing the shielding intervention]

Inputs: components of the intervention

The intervention was complex. There were three core processes relevant to all people advised to shield: the Chief Medical Officers of the four nations reaching agreement on the clinical criteria for inclusion on the CEV list; identification of people to be added to the list; and communication with those identified.

Identifying and communicating with CEV people took place in phases, with batches of individuals being added to the list over the time the shielding programme was 'live' and much smaller numbers being removed. Across the UK, CEV people were identified through searches of centralised databases (which produced the majority of names for the list in Wales), and through primary and secondary care records, in conjunction with doctors’ clinical judgement [11]. This mixed approach was described as '*build the list nationally first and then ask GPs to review*' (Participant 2 Policy Maker). Some GPs and practice managers

For peer review only - http://bmjopen.bmj.com/site/about/guidelines.xhtml

responded to people requesting to be removed from, or added to, the list. As one respondent noted, this was more significant than a mere administrative process:

> '*The GP had the authority to put people on and off, but a lot of the GPs hadn't realised… what the implication of that was for the individual about going back to work, back to school, or actually getting access to the food box or not.*' (Participant 9 Local government)

The development of the QCovid risk prediction tool, which identified clinical and demographic risk factors for COVID-19-related hospitalisations and deaths [12 ,13] led to substantial numbers being added to CEV lists in the summer of 2020.

The 'binary' nature of inclusion on the list was seen by one respondent as problematical:

> *'It didn't reflect the spectrum of risk. So you were selecting a group of people for really quite an austere set of advisory statements, but actually nothing for the rest.' (Participant 1 Policy Maker*)

Letters were sent to CEV people strongly advising them to shield from 23rd March 2020, initially for 12 weeks [14]. Respondents reported considerable thought going into the exact wording of the letters, with an emphasis on them giving advice, not instruction:

> *'We were telling people they could rather than telling people they must. ….It was strong advice, but it was set in advisory terms.' (Participant 7 Policy maker)*

Where telephone numbers were available, reinforcement of the advice was sent by text message.

The other aspect of the intervention was the support programme, a range of components experienced by some CEV people as required. These included eligibility (achieved through a change in legislation) for Statutory Sick Pay for CEV people, even if they were not ill, to allow them to take time off work if their job could not be done at home. For those who needed them, the UK government contracted with commercial providers to deliver weekly food parcels. CEV people could also access delivery of medications and were given priority by supermarkets for food delivery.

Other forms of support were available through local government and partner organisations, and CEV people were invited to register to access this. Welsh local government respondents

For peer review only - http://bmjopen.bmj.com/site/about/guidelines.xhtml

described taking a proactive approach to contacting CEV people who might be in need of help, through welfare telephone calls:

> *'trying to keep [CEV people] linked into their society, their communities. So it was very much a kind of social response to it…Most of the people doing the calling were our librarians.'* (Participant 10 Local government)

Although changes to data sharing rules allowed lists of CEV people to be supplied to local government, significant challenges were reported with using the shared data:

> *'It was incomplete. It wasn't accessible…It wasn't transferable. It didn't match any of the other datasets. We couldn't identify individuals through it. I think the first lot didn't even have people's names. It just had their NHS number.'* (Participant 11 Local government)

Voluntary and community sector (VCS) organisations often worked in co-ordination with local and central government, running telephone helplines offering advice and emotional support for isolated people:

> *'We switched from a Monday to Friday, nine to five operation, to a seven day a week operation. …We were even doing things like contacting very local suppliers, and shops, to see if we could get deliveries put in place for people.'* (Participant 3 Voluntary and community sector)

Predicted mechanisms of change and outcome

The predicted mechanism of change was that people would isolate themselves, with the outcome of avoiding infection. Some respondents expressed anxiety about the loss of liberty this represented, even though '*it was really quite an honourable aim'* (Participant 1 Policy Maker). Initially, shielding people were advised to avoid all contact with others, even within the home. Respondents were aware that this was problematical:

> '*Everybody realised that wasn't realistic for ninety per cent of people, who don't live in houses with west wings and east wings. And so… it then became a household isolation, which was even worse.' (Participant 1 Policy Maker)*

For peer review only - http://bmjopen.bmj.com/site/about/guidelines.xhtml

Equally, some of those shielding would require care at home, with care workers a potential source of infection:

> *'A number of these people were vulnerable, they had comorbidities, they required support to manage that, and therefore contact would occur'. (Participant 5, Policy Maker)*

Respondents were aware that adherence to shielding advice would vary between individuals, and also over time, but felt that overall risk would still be reduced:

> *'It's not an all or nothing, it's not if you break it once, you've broken the law, like with the legislative regulations.' (Participant 2 Policy Maker)*

Intended impact

The primary intended impact was a reduction in mortality among CEV people. This was emphasised strongly by most respondents.

> *'The idea is that if we isolate them they're less likely to get it, because if they get it they're probably going to be really, really poorly and die.' (Participant 5, Policy Maker)*

Some respondents described a broader range of beneficial impacts, including limiting the burden on the NHS, engendering of community spirit, and more social and voluntary support for isolated vulnerable people.

> *'It's about protecting the NHS, because that's in all our interests, isn't it, but actually on a local level, it was very much about supporting our communities.' (Participant 10, Local government)*

Risks/unintended impact

All participants described potential risks or unintended impact of shielding, and in particular the impact which it might have on mental health, through isolation and anxiety:

> *'Some people who are shielding are still able to be effectively engaged somehow with society. They're working from home or whatever. But others have suffered, probably*

For peer review only

For peer review only - http://bmjopen.bmj.com/site/about/guidelines.xhtml

a lot…It created a whole lot of knock-on anxiety and everybody who was related in any way with the shielded person [was] put in a state of heightened awareness and heightened anxiety.' (Participant 1 Policy Maker)

Concerns about the long-term mental health impact were reported as a factor in the decision to pause shielding advice in July 2020:

'The effect on mental health started to outweigh the benefit once the prevalence was low enough.' (Participant 2 Policy Maker)

In addition, concerns were widely expressed about the impact of shielding on physical health, including muscle wastage:

'The debilitation from not leaving the house …this was not just that you were telling ninety- year-olds and eighty-year-olds, you were telling this to often quite fit young people who just had another condition to stay in the house.' (Participant 4, Policy Maker)

Other unintended impacts included that on the workforce, as CEV people whose jobs could not be done from home were no longer available to work.

Contextual factors

The shielding policy was based on the assumption that the CEV group would have enhanced protection over that of the general population. Almost as soon as shielding started, a lockdown was introduced for the wider population, imposing legally mandated restrictions on spending time outside the home except for certain exemptions. The national lockdown also brought certain whole-population initiatives, such as the 'furlough scheme' to subsidise wages for staff on temporary leave.

Respondents reflected that although lockdown was likely to have slowed the spread of the virus in the wider population, continuing steady rates of infection meant that shielding needed to continue to run in parallel:

'It went on an awful lot longer than was envisaged in the first instance.' (Participant 4 Policy Maker)

For peer review only - http://bmjopen.bmj.com/site/about/guidelines.xhtml

Since the shielding policy was developed in the context of a crisis, it - along with the associated shielding programme - was devised and implemented at an unusually fast rate, and participants spoke of a clear shared purpose:

> *'We all had to make decisions differently and quickly, and we all had to use a bit more common sense than the traditional waiting for somebody else to come up with a strategy.' (Participant 8 Local government)*

Local government respondents reported that there was a blurring around the edges of the population receiving their input, as they added people they knew to be vulnerable to their lists for this type of support, in addition to CEV people on the shielding list.

> *'Our proactive calling quickly extended to older people who were not necessarily shielding, but we felt they were also vulnerable – a number of them were... in effect shielding themselves.' (Participant 10 Local government)*

In addition to the formal social support available from local authorities and third sector organisations, many CEV people were likely to have been able to access a wide range of other resources within the community, which enabled shielding to happen, especially informal support from family and friends. However, some would have lacked informal support from family, friends, and neighbours and may have become increasingly isolated.

Discussion

Principal findings

Our logic model provides a visual presentation of our understanding of the programme theory underlying the shielding intervention introduced across the UK to protect the most vulnerable people doing the early phases of the COVID-19 pandemic. It captures the key components of the shielding intervention (policy plus support programme), and identifies the mechanism by which it might make a difference, potential impacts, both unintended and intended, and key contextual factors. The logic model will underpin our evaluation of the impact of the shielding intervention, and we will continue to review it as the evaluation is finalised, in order to provide a structure to evaluating an intervention which to an extent was fluid and extemporised.

For peer review only - http://bmjopen.bmj.com/site/about/guidelines.xhtml

Our interviews with the key stakeholders add three main areas of insight: into the iterative and fluid process of developing and implementing shielding; into the range of activities which local government and the third sector introduced to support shielding people, outside the formal bounds of the programme; and into the feelings of many of those involved in the process, who revealed uncertainty about not only the logic but also the justice of shielding. The shielding policy, like many other aspects of the UK government's response to SARS-CoV-2, was introduced at pace. This left those involved in implementing the policy and delivering the shielding programme to work out the details at speed, once the decision had been made. The policy implementation and support programme were designed and iteratively refined by the many different parties collaborating on the work across the four nations of the UK – not just SAGE, civil servants and clinicians, but also those involved in providing services at local and community level. In the delivery of the shielding support programme, there was some blurring, as the formal shielding policy ended up being delivered alongside support interventions for those regarded as vulnerable for social rather than clinical reasons. This does not undermine the programme theory, but instead is integral to it.

The nature of implementation as a process over time could also be observed. Although the basic principle of shielding remained the same throughout the time period of the intervention, the details evolved significantly – in terms of the nature of the advice, the definition of who should be on the shielding list, and in turn, the numbers of those included. As the pandemic continued, the evolution of the innovative attempts to deal with its impact were obvious, with new ways of working emerging between national government, local government and the voluntary and community sector.

There was some ambiguity about who was doing the shielding: the use of the term 'Shielded Patient List' in guidance from the Department of Health and Social Care [15] implies that those at risk were being shielded by the state; yet the advisory nature of the guidance suggests that people were being asked to actively shield themselves – shielding rather than being shielded.

For peer review only - http://bmjopen.bmj.com/site/about/guidelines.xhtml

Context of other literature

The changes in the intervention over time noted in our study reflect the evolving nature of the shielding list, and the slippage between guidance and advice in public discourse, has been tracked in detail by Herrick [16]. Emerging evidence suggests that, despite the shielding intervention, there were still high rates of infection, hospitalisation and mortality in the shielding group [3,17] casting doubt on the mechanism proposed. This may in part be due to the impracticality of truly isolating people, particularly those who were in contact with clinical care providers and carers due to their vulnerability. Modelling has suggested that under an 'imperfect' but realistic shielding strategy, in which contacts for those shielding were reduced by 80%, would still allow high rates of infection of high risk individuals, with deaths estimated at 150% -300% higher than under an implausible 'perfect' shielding model in which contacts were reduced to zero [18]. High rates of nosocomial COVID-19 infection have been identified, likely to disproportionately affect CEV people [19-21].

Our findings confirm previous studies which have identified the crucial role of local government and VCS organisations in supporting the implementation and operationalisation of the shielding policy [22], and previous work discussing local variation in how the CEV list was created and the proportion and make-up of population on it [1], in particular associated with the addition of people to the list by local clinicians [23]. Communication with CEV people to inform them of the support available has been reported elsewhere as missing thousands of people, as records were incomplete [23]. A rapid evaluation in Scotland concluded that while the principle of shielding was valid, the intervention should not be repeated in exactly the same format [24].

The concerns expressed in our interviews about potential negative impacts of shielding reflect suggestions from other studies that people advised to shield may have experienced increased anxiety and mental ill-health and struggled to access routine healthcare [1 ,25], and there may have been an additional strain on unpaid carers who were left without their usual support [26].

For peer review only - http://bmjopen.bmj.com/site/about/guidelines.xhtml

Strengths and limitations

This is the first study to develop a logic model examining all components of the UK COVID-19 shielding policy and programme, and to report the perspective of those involved in operationalising it.

In this phase of the EVITE Immunity evaluation, we did not record the perspective of CEV people themselves, who will have adhered to a greater or lesser extent, with adherence perhaps changing over time [11]; later phases of EVITE Immunity will explore the experience of this group.

Implications

The UK's response to the pandemic of advising shielding for the most vulnerable was an unusual one, paralleled most closely in Europe by Ireland and by Sweden, where a policy of shielding people aged over 70 contrasted with the general population lock downs of its Nordic neighbours [27]. Although initial modelling in the UK explored a similar community level, age-based approach to shielding [28 ,29], the adoption instead of an approach based on identifying and targeting individual clinical vulnerability was substantially more complex, and perhaps was regarded as more acceptable to the public. The emphasis in the UK on a mitigation approach – reducing the peak of infection while protecting the vulnerable – was soon overtaken by the imposition of general population lockdowns, but the two policy approaches continued to run in parallel, at least initially. Modelling early in the pandemic had identified a potential trade-off between increasing protection of the vulnerable and relaxing restrictions on the non-vulnerable [11]. However, the overlapping lockdown and shielding restrictions in the UK, together with the 'leakage' of shielding through necessary personal contacts, along with rates of full compliance reported as down to less than two thirds of those on the list by early summer 2020 [30] make it hard to measure how this has played out. The impact of shielding will have depended in part on people's willingness to comply with guidance, shaped in turn by media narrative and social norms. There was no equivalent guidance on second level shielding, for those essential close contacts of the shielded population, and measures to control infection within the wider population were imperfect.

For peer review only

For peer review only - http://bmjopen.bmj.com/site/about/guidelines.xhtml

Over time, there has been an evolution of risk within the shielded population resulting from subsequent waves of coronavirus infection, along with the vaccination programme. Shielding has now formally ended in both England and Wales. The most recent government guidance to previously shielding people in England reassured that, with protection from the vaccine, they would no longer be at substantially greater risk than the rest of the population [31], though new mutations challenge the efficacy of existing vaccines. A now much better defined core high risk subset remains, consisting of those unable to respond to vaccination appropriately [32].

In terms of both infection rates and mortality in the UK, the pandemic has been identified as having an unequal impact on the population, reflecting health inequalities ultimately rooted in social inequalities [32]. The shielding policy may have exacerbated these, as those living in more crowded accommodation would have found it more challenging to maintain isolation from others.

Conclusion

The shielding intervention was introduced to save lives by protecting the most vulnerable to SARS-COV-2 infection. The shielding programme of support was introduced particularly rapidly and involved novel collaborations between various agencies. Components varied slightly but were broadly similar across the UK. It was a hitherto largely untested strategy based on 'common sense' risk mitigation rather than evidence based interventions.

Naturally, this large scale initiative created challenges both for those attempting to implement the policy and for those meant to benefit from it. Our logic model allows us to understand the different impacts (intended and unintended) of the shielding programme on organisations and populations, and spells out its rationale, components, and mechanisms. Developing the logic model with input from key stakeholders has given additional insight to help us understand the causal links which will inform our evaluation of the impact of shielding and help us to understand its effect and limitations.

Ethics:

For peer review only - http://bmjopen.bmj.com/site/about/guidelines.xhtml

The EVITE Immunity study has received approval from the Newcastle North Tyneside 2 Research Ethics Committee (IRAS 295050).

Transparency statement:

Alison Porter is the manuscript's guarantor and affirms that the manuscript is an honest, accurate, and transparent account of the study being reported; that no important aspects of the study have been omitted; and that any discrepancies from the study as originally planned (and, if relevant, registered) have been explained.

Contributorship statement:

AP contributed to the conception and design of the study, to data acquisition, analysis and interpretation, and led the drafting of the paper. BE and VW contributed to the conception and design of the study, to data acquisition, analysis and interpretation, and critically reviewed the draft paper. LD and LG contributed to the conception and design of the study, contributed to data analysis and interpretation, and critically reviewed the draft paper. AA, AC-S, JD, AE, AJ, SJ, MK, RL, JM, BS, AW contributed to the conception and design of the study and critically reviewed the draft paper. HS led the study, contributed to the conception and design of the study and critically reviewed the draft paper.

Competing interests:

Ronan Lyons, Stephen Jolles, Ann John and Adrian Edwards are members of the Welsh Government COVID-19 Technical Advisory Group. Ann John is also co-Chair of the Scientific Pandemic Insights Group on Behaviours (SPI-B) which is a subgroup of the Scientific Advisory Group for Emergencies (SAGE) advising the UK government. Stephen Jolles is also a member of the Welsh Government Testing TAG and Cardiff University COVID Strategic Advisory Board (SAB). No other authors have competing interests.

Funding statement:

This work is supported by the National Core Studies Immunity (NCSi4P) Programme.

Data availability statement:

For peer review only

No additional data available.

For peer review only

For peer review only - http://bmjopen.bmj.com/site/about/guidelines.xhtml

References

1. Hodgson K, Butler JE, Davies A, et al. Briefing: Assessing the impact of COVID-19 on the clinically extremely vulnerable population. Health Foundation. 2021
2. Lyons J, Akbari A, Torabi F, et al. Understanding and responding to COVID-19 in Wales: protocol for a privacy-protecting data platform for enhanced epidemiology and evaluation of interventions. *BMJ Open* 2020;10(10):e043010.
3. Snooks H, Watkins A, Lyons J et al. Did the UK's public health Shielding policy protect the clinically extremely vulnerable during the COVID-19 pandemic in Wales? Results of EVITE Immunity, a linked data retrospective study. *Public Health* 2023 in press https://doi.org/10.1016/j.puhe.2023.02.008
4. Evans BA, Akbari A, Bailey R, et al. Evaluation of the shielding initiative in Wales (EVITE Immunity): protocol for a quasiexperimental study. *BMJ Open* 2022;12(9):e059813.
5. Skivington K, Matthews L, Simpson SA, et al. A new framework for developing and evaluating complex interventions: update of Medical Research Council guidance. *BMJ* 2021;374:n2061. doi: 10.1136/bmj.n2061 [published Online First: 20210930]
6. Langley T, Gillespie D, Lewis S, et al. Developing logic models to inform public health policy outcome evaluation: an example from tobacco control. *Journal of Public Health* 2021;43(3):639-46.
7. Ritchie J, Spencer L. Qualitative data analysis for applied policy research. Analyzing qualitative data: Routledge 2002:187-208.
8. National Institute for Health Research. UK Standards for Public Involvement., 2019.
9. Staniszewska S, Brett J, Mockford C, et al. The GRIPP checklist: strengthening the quality of patient and public involvement reporting in research. *Int J Technol Assess Health Care* 2011;27(4):391-9. doi: 10.1017/S0266462311000481
10. Evans BA, Gallanders J, Griffiths L, et al. Public involvement and engagement in primary and emergency care research: the story from PRIME Centre Wales. *International Journal of Population Data Science* 2020;5(3)
11. Davies G. Protecting and supporting the clinically extremely vulnerable during lockdown. London: National Audit Office, Department of Health and Social Care,, 2021.
12. Clift AK, Coupland CAC, Keogh RH, et al. Living risk prediction algorithm (QCOVID) for risk of hospital admission and mortality from coronavirus 19 in adults: national derivation and validation cohort study. *BMJ* 2020;371:m3731. doi: 10.1136/bmj.m3731 [published Online First: 20201020]
13. Lyons J, Nafilyan V, Akbari A, et al. Validating the QCOVID risk prediction algorithm for risk of mortality from COVID-19 in the adult population in Wales, UK. *Int J Popul Data Sci* 2020;5(4):1697. doi: 10.23889/ijpds.v5i4.1697 [published Online First: 20220215]
14. Welsh Government. Guidance on protecting people defined on medical grounds as clinically extremely vulnerable from coronavirus (COVID-19) – previously known as 'shielding'. Information for protecting people defined on medical grounds as extremely vulnerable from COVID-19., 2022.
15. Department of Health and Social Care. Guidance on protecting people who are clinically extremely vulnerable from COVID-19 In:England., 2021.
16. Herrick C. 'We thank you for your sacrifce': Clinical vulnerability, shielding and biosociality in the UK's Covid-19 response. Biosocieties Jan 22;1-23. doi: 10.1057/s41292-021-00266-0
17. Jani BD, Ho FK, Lowe DJ, et al. Comparison of COVID-19 outcomes among shielded and non-shielded populations. *Scientific Reports* 2021;11(1):15278. doi: 10.1038/s41598-021-94630-6
18. Smith CA, Yates CA, Ashby B. Critical weaknesses in shielding strategies for COVID-19. PLOS Glob Public Health, 2022 2(4): e0000298.

19. Ponsford MJ, Jefferies R, Davies C, et al. Burden of nosocomial COVID-19 in Wales: results from a multicentre retrospective observational study of 2508 hospitalised adults. *Thorax* 2021;76(12):1246-49.
20. Ponsford MJ, Ward TJ, Stoneham SM, et al. A systematic review and meta-analysis of inpatient mortality associated with nosocomial and community COVID-19 exposes the vulnerability of immunosuppressed adults. *Frontiers in immunology* 2021;12:744696.
21. McKeigue PM, McAllister DA, Caldwell D, et al. Relation of severe COVID-19 in Scotland to transmission-related factors and risk conditions eligible for shielding support: REACT-SCOT case-control study. *BMC medicine* 2021;19(1):1-13.
22. Bevan Foundation. Lessons from lockdown: the experience of shielding. 2020 [Available from: https://www.bevanfoundation.org/resources/experience-of-shielding/.
23. House of Commons Committee of Public Accounts. COVID-19: supporting the vulnerable during lockdown. Fifty-third report of Session 2019-21. HC938. 2021
24. Public Health Scotland. Covid-19 Shielding Programme (Scotland) Rapid Evaluation: Full Report. Edinburgh: Public Health Scotland, 2021.
25. Fisher A, Roberts A, McKinlay AR, et al. The impact of the COVID-19 pandemic on mental health and well-being of people living with a long-term physical health condition: a qualitative study. *BMC Public Health* 2021;21(1):1-12.
26. Lorenz-Dant K, Comas-Herrera A. The impacts of COVID-19 on unpaid carers of adults with long-term care needs and measures to address these impacts: A rapid review of evidence up to November 2020. *Journal of Long-Term Care* 2022;2021:124-53.
26. Davies NG, Kucharski AJ, Eggo RM, et al. Effects of non-pharmaceutical interventions on COVID-19 cases, deaths, and demand for hospital services in the UK: a modelling study. *The Lancet Public Health* 2020;5(7):e375-e85.
28. Ferguson NM, Laydon D, Nedjati-Gilani G, et al. Impact of non-pharmaceutical interventions (NPIs) to reduce COVID-19 mortality and healthcare demand. 2020
29. Van Bunnik BA, Morgan AL, Bessell PR, et al. Segmentation and shielding of the most vulnerable members of the population as elements of an exit strategy from COVID-19 lockdown. *Philosophical Transactions of the Royal Society B* 2021;376(1829):20200275.
30. Department of Health and Social Care. Guidance for people previously considered clinically extremely vulnerable from COVID-19., 2022.
31. Ponsford MJ, Evans K, Carne EM, et al. COVID-19 Vaccine Uptake and Efficacy in a National Immunodeficiency Cohort. *Journal of Clinical Immunology* 2022;42(4):728-31. doi: 10.1007/s10875-022-01223-7
32. Jung A-S, Haldane V, Neill R, et al. National responses to covid-19: drivers, complexities, and uncertainties in the first year of the pandemic. *BMJ* 2021;375

For peer review only - http://bmjopen.bmj.com/site/about/guidelines.xhtml

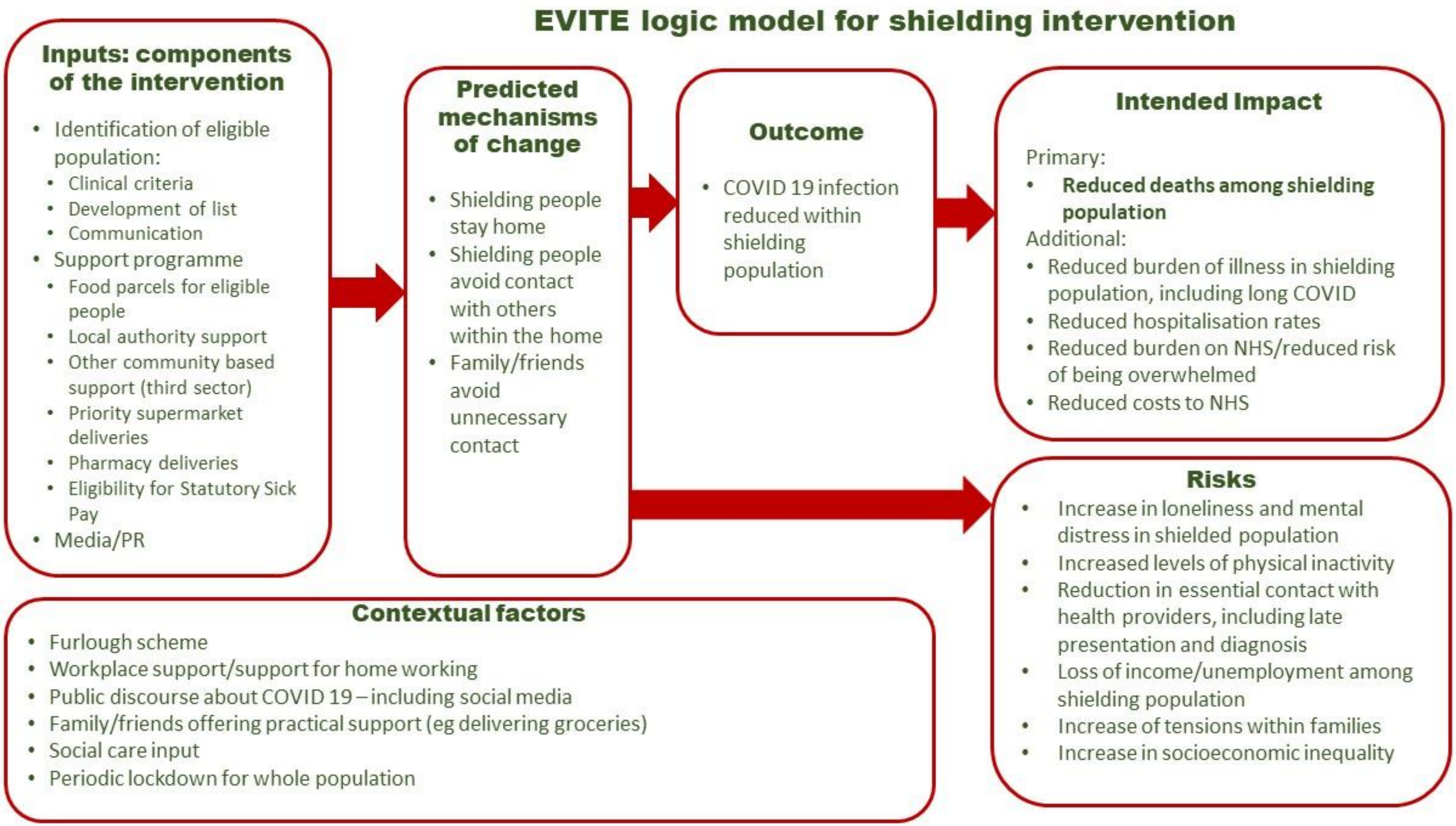
EVITE logic model for shielding intervention
Inputs: components of the intervention
• Identification of eligible population:
• Clinical criteria
• Development of list
• Communication
• Support programme
• Food parcels for eligible people
• Local authority support
• Other community based support (third sector)
• Priority supermarket deliveries
• Pharmacy deliveries
• Eligibility for Statutory Sick Pay
• Media/PR
Predicted mechanisms of change
• Shielding people stay home
• Shielding people avoid contact with others within the home
• Family/friends avoid unnecessary contact
Outcome
• COVID 19 infection reduced within shielding population
Intended Impact
Primary:
• Reduced deaths among shielding population
Additional:
• Reduced burden of illness in shielding population, including long COVID
• Reduced hospitalisation rates
• Reduced burden on NHS/reduced risk of being overwhelmed
• Reduced costs to NHS
Risks
• Increase in loneliness and mental distress in shielded population
• Increased levels of physical inactivity
• Reduction in essential contact with health providers, including late presentation and diagnosis
• Loss of income/unemployment among shielding population
• Increase of tensions within families
• Increase in socioeconomic inequality
Contextual factors
• Furlough scheme
• Workplace support/support for home working
• Public discourse about COVID 19 – including social media
• Family/friends offering practical support (eg delivering groceries)
• Social care input
• Periodic lockdown for whole population

Appendix **EVITE Immunity Phase 1 – WP1 QUESTIONS FOR STAKEHOLDER CONSULTATION**

This consultation is about understanding the rationale for the shielding programme in Wales for patients clinically extremely vulnerable to COVID-19 infection.

1. What was the objective of the shielding programme?
    - To reduce mortality among vulnerable people
    - To limit pressure on the NHS
    - To reduce infection rates/morbidity
    - Were there multiple objectives? If so, was one more important that the others?
    - Did the objectives change over time?

2. How was the shielding programme designed to work?
    - Change in behaviour of people at risk (staying home/avoiding contact)
    - Change in behaviour of other household members (staying home/avoiding contact)
    - Adaptations made by employers (allowing people to work from home, furloughing staff)
    - Increasing awareness of those clinically vulnerable aka the shielding population
    - Increasing awareness of risk among the wider public
    - Was there any change over time in how the programme was designed to work
3. What was needed to make it work?
    - Identifying people at risk – datasets, risk algorithms, clinical judgement
    - Information/communication with people on the list (letter vs text mechanism and accessibility of these)
    - Support/advice from medical staff (GPs or consultants managing patients' conditions)
    - Support/advice from carers of those identified as clinically vulnerable
    - Food parcels
    - Benefits/compensation for people unable to work
    - Have all these been in place? Has anything changed over time?

4. How was the shielding programme developed?
    - Where did the idea come from?
    - Who was involved in developing the programme?
    - Who made the decisions?
    - What contact was there with partners across the UK?
    - How did the shielding programme in Wales relate to England?
    - as there consensus among all involved about the objectives of the programme and what form it should take?

5. How has the shielding programme been reviewed/monitored?
    - What information has been gathered?
    - What has changed as a result?

For peer review only - http://bmjopen.bmj.com/site/about/guidelines.xhtml

Standards for Reporting Qualitative Research (SRQR)*

http://www.equator-network.org/reporting-guidelines/srqr/

Title and abstract

	Page/line no(s).
**Title** - Concise description of the nature and topic of the study Identifying the study as qualitative or indicating the approach (e.g., ethnography, grounded theory) or data collection methods (e.g., interview, focus group) is recommended	P1
**Abstract** - Summary of key elements of the study using the abstract format of the intended publication; typically includes background, purpose, methods, results, and conclusions	P2

Introduction

**Problem formulation** - Description and significance of the problem/phenomenon studied; review of relevant theory and empirical work; problem statement	P4
**Purpose or research question** - Purpose of the study and specific objectives or questions	P4

Methods

**Qualitative approach and research paradigm** - Qualitative approach (e.g., ethnography, grounded theory, case study, phenomenology, narrative research) and guiding theory if appropriate; identifying the research paradigm (e.g., postpositivist, constructivist/ interpretivist) is also recommended; rationale**	P5
**Researcher characteristics and reflexivity** - Researchers' characteristics that may influence the research, including personal attributes, qualifications/experience, relationship with participants, assumptions, and/or presuppositions; potential or actual interaction between researchers' characteristics and the research questions, approach, methods, results, and/or transferability	P5
**Context** - Setting/site and salient contextual factors; rationale**	P5
**Sampling strategy** - How and why research participants, documents, or events were selected; criteria for deciding when no further sampling was necessary (e.g., sampling saturation); rationale**	P5
**Ethical issues pertaining to human subjects** - Documentation of approval by an appropriate ethics review board and participant consent, or explanation for lack thereof; other confidentiality and data security issues	P15
**Data collection methods** - Types of data collected; details of data collection procedures including (as appropriate) start and stop dates of data collection and analysis, iterative process, triangulation of sources/methods, and modification of procedures in response to evolving study findings; rationale**	P5

For peer review only

**Data collection instruments and technologies** - Description of instruments (e.g., interview guides, questionnaires) and devices (e.g., audio recorders) used for data collection; if/how the instrument(s) changed over the course of the study	P5
**Units of study** - Number and relevant characteristics of participants, documents, or events included in the study; level of participation (could be reported in results)	P5
**Data processing** - Methods for processing data prior to and during analysis, including transcription, data entry, data management and security, verification of data integrity, data coding, and anonymization/de-identification of excerpts	P5
**Data analysis** - Process by which inferences, themes, etc., were identified and developed, including the researchers involved in data analysis; usually references a specific paradigm or approach; rationale**	P5
**Techniques to enhance trustworthiness** - Techniques to enhance trustworthiness and credibility of data analysis (e.g., member checking, audit trail, triangulation); rationale**	P5

Results/findings

**Synthesis and interpretation** - Main findings (e.g., interpretations, inferences, and themes); might include development of a theory or model, or integration with prior research or theory	Pp6-11
**Links to empirical data** - Evidence (e.g., quotes, field notes, text excerpts, photographs) to substantiate analytic findings	Pp6-11

Discussion

**Integration with prior work, implications, transferability, and contribution(s) to the field -** Short summary of main findings; explanation of how findings and conclusions connect to, support, elaborate on, or challenge conclusions of earlier scholarship; discussion of scope of application/generalizability; identification of unique contribution(s) to scholarship in a discipline or field	Pp11-13
**Limitations** - Trustworthiness and limitations of findings	P14

Other

**Conflicts of interest** - Potential sources of influence or perceived influence on study conduct and conclusions; how these were managed	P16
**Funding** - Sources of funding and other support; role of funders in data collection, interpretation, and reporting	P15

*The authors created the SRQR by searching the literature to identify guidelines, reporting standards, and critical appraisal criteria for qualitative research; reviewing the reference lists of retrieved sources; and contacting experts to gain feedback. The SRQR aims to improve the transparency of all aspects of qualitative research by providing clear standards for reporting qualitative research.

For peer review only

**The rationale should briefly discuss the justification for choosing that theory, approach, method, or technique rather than other options available, the assumptions and limitations implicit in those choices, and how those choices influence study conclusions and transferability. As appropriate, the rationale for several items might be discussed together.

Reference:

O'Brien BC, Harris IB, Beckman TJ, Reed DA, Cook DA. **Standards for reporting qualitative research: a synthesis of recommendations.** *Academic Medicine*, Vol. 89, No. 9 / Sept 2014 DOI: 10.1097/ACM.0000000000000388

For peer review only

For peer review only - http://bmjopen.bmj.com/site/about/guidelines.xhtml
